# A Review on Improving the Oxidative Stability of Pine Nut Oil in Extraction, Storage, and Encapsulation

**DOI:** 10.3390/antiox14060716

**Published:** 2025-06-12

**Authors:** Jingwen Zhu, Zhenzhou Li, Yisen Wang, Zhexuan Mu, Xiaohong Lv, Zhenyu Wang, Aijun Dong, Ziluan Fan, Hua Zhang

**Affiliations:** 1 Department of Food Science and Engineering, College of Life Sciences, Northeast Forestry University, Harbin 150040, China; gwen1299@163.com; 2 Department of Special Food and Drug and Biochemical Innovation Research Center, School of Chemistry and Chemical Engineering, Harbin Institute of Technology, Harbin 150001, China; 3 Heilongjiang Academy of Forestry Sciences, Harbin 150040, China

**Keywords:** pine nut oil, unsaturated fat, lipid oxidation, antioxidant strategies, green technologies, storage stability, preventive measures

## Abstract

Pine nut oil (PNO) is highly valued by consumers for its rich content of unsaturated fatty acids, which confer unique nutritional benefits. However, PNO is highly susceptible to lipid oxidation during storage and extraction. This chemical degradation compromises product quality and poses potential risks to food safety. To address this challenge, the food industry is developing antioxidant strategies, including optimizing pretreatment conditions to improve flavor and storage stability. Green extraction technologies such as microwave-assisted extraction (MAE) and ultrasonic-assisted extraction (UAE) have been introduced to enhance extraction efficiency and promote environmental sustainability. Light-proof packaging, reduced oxygen environments, and temperature control have also been employed to significantly extend the shelf life of PNO. Furthermore, to maintain the nutritional integrity and safety of PNO while expanding its functional applications in the food industry, several innovative approaches have been employed. These include the incorporation of natural antioxidants, the development of Pickering emulsions, the use of microencapsulation, and the formulation of oleogels.

## 1. Introduction

Pine nuts belong to the genus *Pinus* [1,2], with 29 species identified as edible. The most commonly consumed pine nuts include *Pinus koraiensis* (Korean pine), *P. sibirica* (Siberian pine), *P. pinea* (stone pine), and *P. gerardiana* (chilgoza pine) [3]. Due to its distinctive nutritional profile and fatty acid (FA) composition, pine nuts are considered a health food in China and Japan. The unique aroma and flavor of pine nuts have become valuable assets in international trade and are widely used in desserts, sauces, and salads [4,5]. Pine nut oil (PNO) is the main component of pine nuts [6]. Unlike most nut oils, which are primarily composed of monounsaturated fatty acids (MUFAs) [7], PNO is rich in polyunsaturated fatty acids (PUFAs), with linoleic acid comprising 47.6% of the total fatty acids [2,8]. Notably, PNO contains a distinct compound: pinolenic acid [9]. These fatty acids confer a wide range of potential applications for PNO in nutritional health care, including hypoglycemic, hypolipidemic, anti-inflammatory, and antioxidant effects [10].

Global pine nut production currently stands at approximately 20,000 tonnes per year, with Turkey leading worldwide production and fully commercializing PNO [11]. Plant-based unsaturated fatty acids (UFAs) have promising potential as alternatives to marine-derived fatty acids. The demand for pine nuts and PNO in the global market continues to rise, primarily due to increased health awareness and dietary diversification [8,9]. The Indian market for nutrition and nutraceuticals is projected to grow from USD 3 billion to USD 6.1 billion by 2022. The growth is largely driven by the increasing trend toward plant-based diets, which boosts demand for healthy vegetable oils [12]. As consumer focus on functional ingredients increases, PNO with its high UFA content, exceeding 90%, has become a key link between the traditional pine nut industry and the modern healthy consumer market. It adds value to the industry through further processing, including dietary supplements and plant-based dairy substitutes.

**Figure 1:**
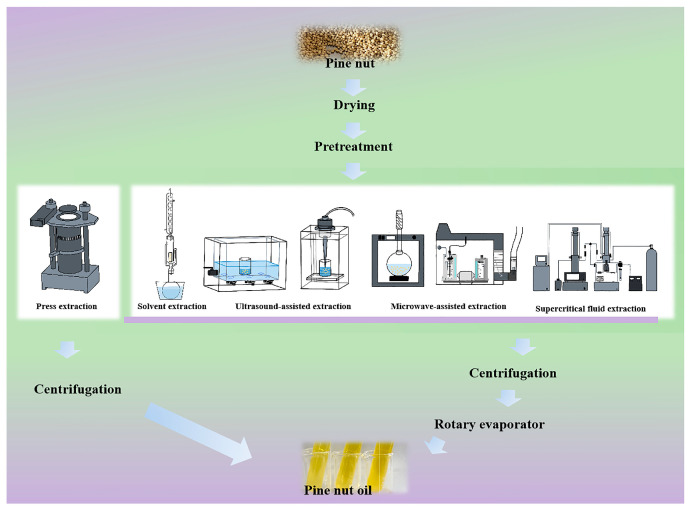
Flow chart of the extraction of PNO.

Despite the growing use of fatty acids in food production [13], PNO remains susceptible to chemical and microbiological contamination, as well as risks of lipid peroxidation similar to those of pecans, red meat, and certain fish [14,15,16]. Additionally, hazardous by-products may be generated during processing [17]. Even under regulated processing and storage conditions, these risks are difficult to eliminate entirely [18,19], which limits the scope of PNO applications in the food industry [20,21]. Current research primarily focuses on the FA composition of PNO and its health effects, while insufficient attention has been given to the quality control of PNO during processing. In contrast, this review systematically examines advanced extraction techniques aimed at improving PNO quality (Figure 1) and explores novel technologies to reduce PNO oxidation.

In this review, a literature search was performed on papers from the Web of Science, Elsevier ScienceDirect, and Wiley Online Library databases, focusing on the period from 2010 to 2025. Initially, the search focused on titles, abstracts and keywords such as “*Pinus*”, “pine”, “oil”, “nut”, “oxidation” “extraction”, “pinolenic”, “storage”, “oleogel”, “emulsion”, “microencapsulation”, and “antioxidant”, resulting in 834 papers. EndNote X9.1 citation management software was used to remove exact duplicates based on title, author, year of publication, and DOI. Non-English articles unrelated to the research question were excluded after reviewing titles and abstracts. A total of 196 papers were identified. Finally, 120 papers met the inclusion criteria after a full text assessment.

## 2. PNO Composition and Lipid Oxidation

Pine nuts are primarily found in Pakistan, China, and Spain. Planted pine forests in Asia and the Americas account for 82% of the global planted area [1,4,22]. The oil yield per 100 g of pine nuts ranges from 45 to 65 g, depending on the method of extraction [3,7,8]. Table 1 presents the FA composition of PNO in different geographical regions. The composition is predominantly high in linoleic acid [23], followed by oleic acid. The ratio of these two fatty acids is typically maintained at over 2:1, which is consistent across most studies [24]. Despite potential germplasm differences among different pine nuts species [25,26], the core fatty acid ratio remains significantly stable. Pinolenic acid is a unique omega-6 PUFA [27,28], and its content varies significantly among pine species [26]. *P. halepensis* Mill. and *P. pinea* L. growing in Tunisia exhibit genetically different levels of pinolenic acid even in the same region [25]. These levels may also be influenced by geographical and climatic conditions. The FA composition of *Pinus pinea* L. from three different geographical regions in Chile, while similar in overall chemical composition to that of the Mediterranean region, varied in lipid content as the mean minimum temperature decreased [7]. Available studies further confirm that genetic factors predominantly influence the variation in pine nut composition [25]. However, geographic distribution and climatic conditions still modulate lipid metabolism to some extent [29].

PNO is rich in several important components, including pinolenic acid, linoleic acid, arachidonic acid, γ-linolenic acid, and α-linolenic acid [10,30], all of which contribute to the prevention of cardiovascular and other chronic diseases. Among these, pinolenic acid not only has potential health benefits, such as lowering cholesterol and reducing elevated blood pressure [31], but also exhibits a wide range of biological activities, including antioxidant, anti-inflammatory, hypolipidemic, anti-atherosclerotic, antidiabetic, and lipid-lowering, as well as inhibition of cancer cell invasion and motility [27,28,32]. However, the realization of these health effects is highly dependent on the quality of PNO, which imposes special demands on processing. Acid value (AV) and peroxide value (PV), as key quality indicators, reflect the free fatty acid content (degree of hydrolytic rancidity) and the accumulation of initial oxidation products, respectively. These values directly affect the freshness and safety of the oil [8,33]. Although the cold pressing process can retain antioxidant substances such as polyphenols and vitamin E, the high content of PUFA still leads to the deterioration of PNO under high temperature or light conditions [34,35].

Oxidation of edible oils is a complex process primarily due to the oxidative susceptibility of the double bonds in PUFA, particularly at the sn-2 position, which is prone to free radical attack [34]. A series of primary and secondary oxidation products generated during the oxidation may affect the taste and flavor of PNO, as well as reduce its nutritional value and health benefits [13]. These changes directly influence the quality and consumer acceptance of the product. Inhibition of lipid oxidation reactions remains a challenge in food science [20]. Lipid oxidation, based on the reaction mechanism and involved factors, can be classified into autoxidation, photooxidation, and enzyme-catalyzed oxidation. Of these, autoxidation is the most common form and one of the spontaneous processes leading to lipid oxidation [8,36]. It consists of initiation, propagation, and termination phases [34].

In the early stages of oxidation, the positions of the double bonds in FA within the triglyceride molecule may shift, forming conjugated dienes and trienes, which mark the onset of the oxidation reaction [37,38]. When UFAs come into contact with oxygen, primary products such as hydroperoxides are formed via the free radical chain mechanism [38]. Although tasteless and odorless, these products affect the quality of PNO. Factors such as temperature and time during PNO extraction significantly influence its PV [8]. Additionally, the greater the number of double bonds in a UFA, the more types of hydroperoxides are formed. Therefore, it is essential to maintain the freshness and stability of PNO. Prolonged high-temperature extraction further accelerates oxidation, causing unstable hydroperoxides to decompose into various secondary oxidation products, such as ketones and aldehydes [33]. This marks the transition to the second stage of lipid oxidation [8]. These volatile products, even in trace amounts, are easily detectable and impart rancidity and off-flavors to the oils, thereby affecting consumer acceptance. They not only alter the physicochemical properties of the oil but also enhance its UV absorption capacity, thereby impairing quality [8,38]. Therefore, controlling the degree of oxidation and minimizing secondary oxidation products is crucial for maintaining PNO quality and consumer acceptance [39].

**Table 1:** Selected data on major fatty acids of pine nuts from different geographical regions.

Geographical Regions	Species	Characteristics of Chemical Structure FA (% of Total FA)			Product Quality	References
		C18:1 Oleic Acid	All-cis-9,12–18:2 Linoleic Acid	All-cis-5,9,12–18:3 Pinolenic Acid		
Xiaoxinganling, Heilongjiang Province, Northeast China	*Pinus koraiens*	25.36	47.67	14.19	/	[10]
Heilongjiang Province, China	*Pinus* *pumila*	27.47	45.18	16.94	The total phenol content: 1.12 mg/g, AV: 2.88 mg KOH/g, PV: 5.25 mmol/kg.	[40]
Eumseong, Korea	*Pinus koraiensis*	26.00	46.00	13.00	/	[30]
The forests of eastern Afghanistan	*Pinus gerardiana* Wall	/	/	19.00	Manganese content: 8.80 mg/100 g.	[41]
Chilean North 31~33° latitude	*Pinus pinea* L.	40.92	47.64	0.37	A-tocopherol: 94.40 μg/kg, γ-tocopherol: 1110.50 μg/kg, total phenolic compounds: 0.27 mg GAE/g.	[7]
Chilean Dry Coast 34~35° latitude	*Pinus pinea* L.	37.13	50.72	0.37	A-tocopherol: 57.40 μg/kg, γ-tocopherol: 1346.90 μg/kg, total phenolic compounds: 0.39 mg GAE/g.	[7]
Chilean South 36~38° latitude	*Pinus pinea* L.	40.28	48.01	0.39	A-tocopherol: 25.30 μg/kg, γ-tocopherol: 756.10 μg/kg, total phenolic compounds: 0.35 mg GAE/g.	[7]
Carregal do Sal	*Pinus pinea* L.	37.59	47.70	0.80	/	[26]
North Algeria Djurdjura National Park located in Tikjda in the state of Bouira	*Pinus halepensis* Mill.	24.55	59.25	/	AV: 68.99 mg KOH/g, PV: 28.69 mmol/kg.	[24]
North Algeria the National Park of Chrea located in the state of Blida	*Pinus pinea* L.	34.63	30.67	/	AV: 19.63 mg KOH/g, PV: 9.95 mmol/kg.	[24]
North Algeria the National Park of Chrea located in the state of Blida	*Pinus pinaster*	18.42	51.95	/	AV: 7.26 mg KOH/g, PV: 67.42 mmol/kg.	[24]
North Algeria the Taza National Park located in the state of Jijel	*Pinus canariensis*	17.43	56.75	/	AV: 4.91 mg KOH/g, PV: 65.88 mmol/kg.	[24]
Algeria	*Pinus halepensis* Mill.	23.95	57.33	/	Contains higher levels of carotenoids, PV: 0.50 mmol/kg.	[23]
Turkey	*Pinus halepensis* Mill.	24.62	57.34	/	PV: 0.79 mmol/kg.	[23]
Tunis	*Pinus halepensis* Mill.	6.70	66.60	/	The EC_50_ value of PNO: 0.35 mM.	[29]

Notes: PV—peroxide value; AV—acid value; GAE—gallic acid equivalent.

## 3. Processing of PNO

The oxidative stability of PNO is a critical quality indicator. It is affected by processing methods, storage conditions, and the characteristics of minor components in the oil [42,43]. Adverse weather and improper processing conditions can lead to fungal contamination of pine nuts. To ensure the high quality of PNO, the oil pressing process should be optimized, storage conditions strictly controlled, and nuts properly preserved. A comprehensive understanding of the influencing factors is essential to maintain the overall quality and nutritional value of PNO [16].

### 3.1. Pretreatment of Pine Nuts

Prior to PNO extraction, appropriate pretreatment is typically performed to improve storage stability and enhance flavor characteristics [44,45,46]. The main preprocessing steps include drying and shelling. Roasting is a commonly used drying method [47]. Roasting reduces the moisture content, enzyme activity, and microbial load of pine kernels through thermal effects [46,47]. It delays fat hydrolysis, lowers aflatoxin levels, and enhances the safety of the oil [48]. Physically, roasting increases the brittleness and hardness of pine nuts, promoting cellular rupture during mechanical pressing. This structural change lowers oil viscosity and surface tension, thereby increasing the oil yield [49]. Roasting at 180 °C for 30 min increased the oil yield from 56.1% to 72.83% and raised the SFA content [6]. At 180 °C for 20 min, a slight increase in MUFA content was observed, while PUFA content declined [49]. This change may result from thermal degradation, as fatty acids with higher unsaturation are more susceptible to heat-induced loss [44]. Roasting may promote the release of bioactive components into the oil, thereby enhancing PNO stability. Roasting at 180 °C for 10 min significantly increased tocopherol and polyphenol contents in PNO [6]. This is not only improved oxidative stability but also enhanced aromatic compound formation, oil flavor, and consumer acceptance [44]. Similar quality improvements have also been observed in other oil-bearing crops such as pumpkin seeds, rapeseed, and walnut kernels [6,46]. These improvements contribute to maintaining oil stability and nutritional value, thereby extending shelf life. This offers an effective strategy for prolonging the shelf life of oil and fat products. Notably, the positive effect of roasting on oil quality is highly dependent on precise control of temperature and time. High temperatures can accelerate lipid oxidation and degrade some antioxidant components. However, they also promote the Maillard reaction, producing compounds with antioxidant activity that improve oil stability [50]. PNO roasted at 150 °C for 30 min exhibited the highest oxidative stability and free radical scavenging capacity. This may be attributed to newly formed Maillard reaction products, elevated total phenolic content, and the presence of residual bioactive components [45]. However, the roasting process must be carefully optimized. Excessive roasting may temporarily raise total phenolic content. However, it also causes significant degradation of photosensitive components, such as carotenoids and chlorophylls. In additional, tocopherol breakdown and loss of other active components may reduce antioxidant capacity and lead to the formation of undesirable flavor compounds [4,6]. Ultimately, over-roasting reduces the nutritional value of the oils [44,45].

### 3.2. Extraction Methodologies of PNO

Selecting the optimal extraction method is essential for maintaining the nutritional value and marketability of oils [8,51]. Different extraction methods and processing intensities influence the chemical composition and antioxidant properties of oils [52]. In addition to press and solvent extraction, advanced technologies offer more efficient and environmentally sustainable alternatives (Table 2).

**Table 2:** PNO extraction methodologies.

Extraction Methodologies	Reasons for Oxidation	Extraction Conditions	Oil Yield (%)	Comment	References
Solvent extraction	High temperature, long time.	Extraction temperature 39.3 °C, extraction time 33.4 min, solvent/sample 10.8:1 (mL g^−1^).	80.03	PV: 0.53 mmol/kg, DPPH-HF: 8.60%, DPPH-LF: 65.07%, DPPH-oil: 73.35%, TV: 3.02.	[8]
Press extraction	High temperature of hot pressing	/	15.50~20.67	PV: 0.50~0.79 mmol/kg, the total amount of tocopherol: 309.42–318.04 mg/100 g, L* values: 92.24~96.04, b* values: 23.47~30.33.	[23]
Ultrasound-assisted extraction	Oxygen, metal probes, high temperature.	2.5% of the enzyme solution mixture, 7.8 mL/g liquid-solid ratio, 1500 rpm stirring speed, 600 W, 50 °C, 1.7 h.	31.89	The total phenol content: 1.12 mg/g, PV: 5.25 mmol/kg.	[40]
Microwave-assisted extraction	High temperature, long time.	Microwave power 420 W, temperature 75 °C, liquid-solid ratio 7.0 mL/g and time 55 min.	24.12	Total phenols: 92,53 mg GAE/kg, α-tocopherol: 315.27 mg/kg, beta-carotene: 25.43 mg/kg, phytosterols: 685.68 mg/kg.	[53]
Supercritical fluid extraction	Oxygen, high temperature.	5760.83 PSI, 50 °C and 3.0 h.	45.85	More unsaturated fat, alpha-linolenic acid, total phenols and flavonoids.	[31,54]

Notes: DPPH: 1,1-Diphenyl-2-picrylhydrazyl; DPPH-HF: DPPH-hydrophilic fraction; DPPH-LF: DPPH-lipophilic fraction; TV: totox value; PV: peroxide value; GAE: gallic acid equivalent.

#### 3.2.1. Solvent Extraction

**Figure 2:**
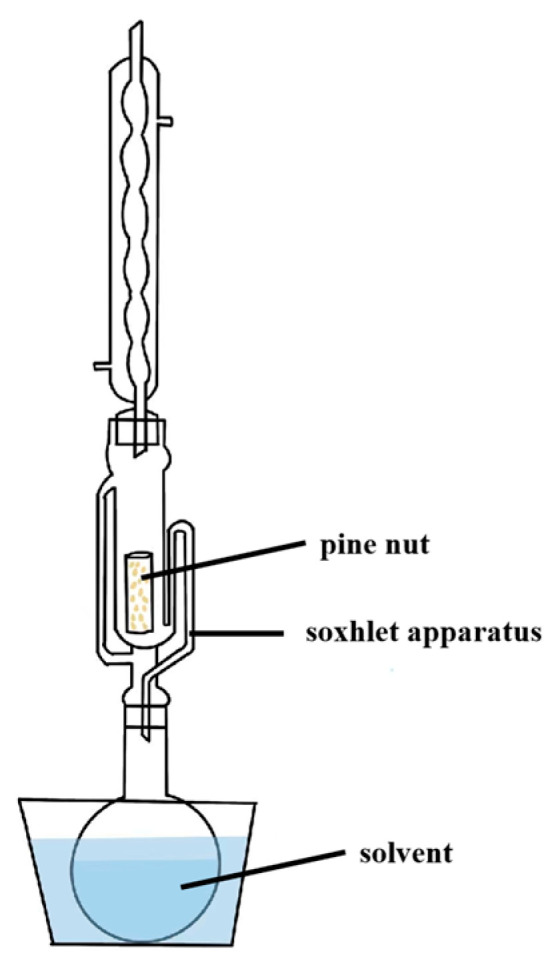
Solvent extraction unit. (Temperature, time, sample moisture content, particle size, and liquid/solid ratio all affect extraction efficiency. The choice of solvent type is particularly critical).

Solvent extraction is widely used in oil processing, particularly for recovery residual oil from low oil content seeds (<20%) and pressed cakes [47], due to its high efficiency and rapid oil recovery [55]. However, the intensive and volatile use of solvents increases production costs and generates toxic waste, posing environment risks [40,56,57]. A schematic of the solvent extraction unit is presented in Figure 2.

Extraction was performed using three open glass flasks. Solid impurities were removed by filtration through filter paper. The solvent was then evaporated under vacuum at 40 °C. The optimal conditions for producing PNO by solvent extraction were extraction temperature 39.3 °C, extraction time 33.4 min, solvent/sample 10.8:1 (mL g^−1^), and extraction efficiency of 80.03%. The extracted oil showed a PV of 0.53 mmol/kg, DPPH-HF of 8.60%, DPPH-LF of 65.07%, and DPPH-oil of 73.35%. A total oxidation value of 3.02 indicated that the oil obtained under these conditions exhibited good oxidative stability [8]. In PNO extraction, factors such as temperature, extraction time, sample moisture content, particle size, and solvent-to-sample ratio significantly affect the extraction efficiency. The type of solvent is also a critical factor in determining extraction outcomes [55]. Traditionally, n-hexane and petroleum ether have been commonly used as solvents for PNO extraction [40]. Although the yield with n-hexane is slightly lower than that of other solvents, its favorable chemical properties and high selectivity result in higher oil clarity and refinement [8]. Notably, extraction efficiency depends not only on the type of solvent but also on material pretreatment. During sample preparation, grinding raw materials into smaller particles significantly increases their surface area, thereby enhancing the contact between the solvent and the sample [55].

The yield obtained through n-hexane extraction (25.38%~28.24%) is higher than that from cold pressing (15.50%~20.67%). However, PNO produced by cold pressing exhibits a lower peroxide value, possibly due to oxidative reactions induced by air exposure during solvent extraction [23]. Although solvent extraction provides high efficiency, the oil quality is generally inferior to that of pressed oil [8,55]. This difference becomes more pronounced at elevated temperatures. Elevated temperatures accelerate the oxidative degradation of UFA in solvent-extracted PNO [53,58]. They also promote the formation of harmful substances and result in the loss of natural antioxidants [58,59]. Notably, the degree of PNO oxidation is positively correlated with both temperature and extraction time. The yellowness index ranges from 33.13 to 62.90 and increases accordingly [8].

To enhance both PNO extraction efficiency and oil quality, a comprehensive consideration of mass and heat transfer is required [53]. Extraction conditions should be optimized, and fine adjustments to each parameter can help achieve the dual goals of high efficiency and superior oil quality.

#### 3.2.2. Press Extraction

**Figure 3:**
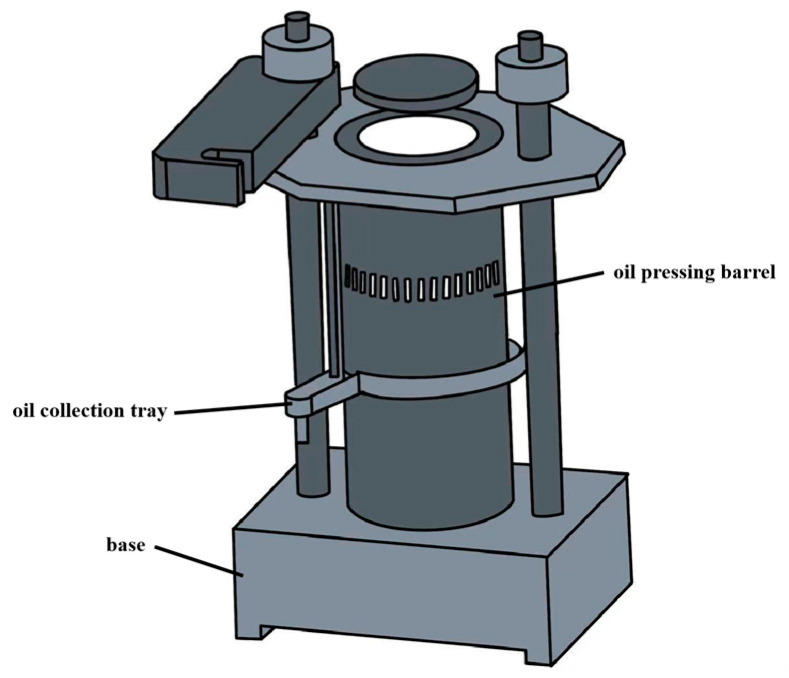
Hydraulic press device. (Important parameters affecting the yield of PNO are the moisture content, temperature, pressure, and particle size of the sample. Higher temperature and pressure and smaller particle size can significantly improve the extraction efficiency of PNO).

Pressing is a solvent-free oil extraction technology [55,60]. It is classified into cold pressing and hot pressing. Cold-pressed edible oils are obtained through mechanical means, such as helical or hydraulic presses, at room temperature [61]. A hydraulic pressing apparatus is illustrated in Figure 3. In contrast, hot pressing increases oil yield by heating pretreated raw materials. Notably, although high temperatures can rapidly disrupt cell walls and reduce oil viscosity, hot pressing may trigger lipolysis and oxidation reactions. These reactions result in higher levels of free FA and peroxides, which significantly differ from those produced during cold pressing [55,62]. Despite differences in processing, both cold-pressed and hot-pressed oils meet the hygienic standards for edible vegetable oils in China, as indicated by their AV and PV [52]. Cold-pressed PNO is rich in tocopherols, polyphenols, and essential FAs. Its PV (0.50~0.79 mmol/kg) is significantly lower than that of solvent-extracted PNO (2.99~3.29 mmol/kg) [60,62]. These findings are consistent with colorimetric analysis, in which the b* values (yellow–blue axis) of pressed oils ranged from 23.47 to 30.34. The L* values (lightness) ranged from 92.24 to 96.04, which were higher than those of solvent-extracted oils (80.82 to 86.53), indicating that pressed oils had greater brightness. The reduction in L* values caused by oxidation is more pronounced in solvent-extracted oils [23]. From a nutritional and physiological perspective, cold-pressed oils are preferred over refined oils [63]. However, their oil yield is usually below 70% [54,57], resulting in a high residual oil content in the press cake, which limits their commercial viability. Nevertheless, cold pressing eliminates the need for energy-intensive solvent treatment and significantly reduces the environmental impact [58]. Therefore, it is considered a preferred method for PNO extraction in resource-constrained regions [55].

Key parameters that influence PNO yield include sample moisture content, temperature, pressure, and particle size. Higher temperatures and pressures, along with smaller particle sizes, significantly enhance the extraction efficiency of PNO [55]. To further improve oil quality and extraction efficiency, cold-pressed PNO is often combined with solvent extraction [44,57,64]. In addition, to overcome the limitations of traditional processes, equipment innovation has become a key focus of development. The development of a novel screw press that eliminates oxygen or uses inert gas during cold pressing can effectively reduce the oxidative degradation of phenolic compounds and improve oil quality [63].

#### 3.2.3. Ultrasound-Assisted Extraction

**Figure 4:**
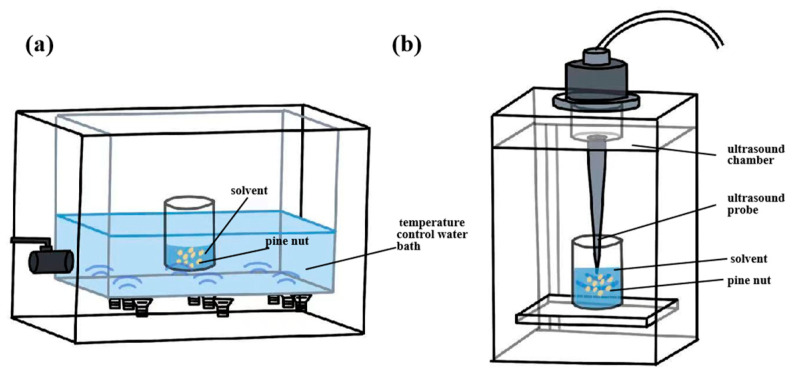
Ultrasonic bath (**a**) and ultrasonic probe (**b**). (Important parameters affecting the yield of PNO are the sonication power, the solvent-to-sample ratio, and the extraction temperature).

Ultrasound-assisted extraction (UAE) has gained increasing attention in recent years for seed oil extraction [56]. An ultrasound-assisted extraction apparatus is presented in Figure 4. UAE relies on ultrasound-induced cavitation and oscillations, which disrupt plant cell walls and facilitate lipid release [40,58]. Increasing ultrasound power has been shown to improve PNO extraction efficiency, mainly due to the intensified cavitation effect. Compared with conventional methods, UAE enhances mass transfer and metabolite release [65], facilitates molecular interactions with solvents at low temperatures [58], protects thermally sensitive components, improves extraction efficiency, and preserves the chemical composition of oils [65].

The yield of UAE is comparable to that of traditional Soxhlet extraction (SE). However, UAE produces oil of superior quality. The PV of PNO obtained by UAE (5.25 mmol/kg) was significantly lower than that from SE (6.16 mmol/kg), while the total phenolic content was higher (1.12 ± 0.03 mg/g) [40]. These results indicate that UAE better retains bioactive components. The antioxidant and physiological properties of these compounds contribute to enhanced oxidative stability and extended shelf life [66]. In addition, UAE shortens the extraction time, reduces degradation, and lowers solvent consumption in rapeseed oil processing [65]. This aligns with current industrial demands for sustainable and health-conscious products [56]. A novel method was developed that integrates homogenization, cyclic ultrasound, and aqueous enzymatic extraction for PNO recovery. The optimized conditions included 2.5% enzyme solution, a 7.8 mL/g liquid/solid ratio, 1500 rpm stirring speed, 600 W ultrasound power, 50 °C temperature, and 1.7 h extraction time. Under these conditions, the extraction yield reached 31.89% [40]. Compared to conventional solvent extraction at high temperatures and prolonged durations, this combined method significantly reduces oil oxidation. Notably, suboptimal values for parameters such as ultrasound amplitude, solvent-to-solid ratio, and extraction temperature can negatively affect yield and quality [55,67]. While moderate ultrasound power enhances mass transfer, excessive intensity increases cavitation, disrupts the molecular structure of oilseeds, and reduces extraction efficiency [68]. A particular concern is the formation of hydroperoxides and lipid oxidation caused by oxygen exposure and interactions with metal probes, which increase peroxide values and conjugated diene levels during UAE [65]. Moreover, ultrasound waves attenuate significantly during medium propagation [68], which limits their effective range and complicates equipment design. Additionally, ultrasonic equipment generates considerable noise during operation [66], which may adversely affect operators and the surrounding environment. Although noise-reduction measures such as acoustic enclosures can be applied, they may raise overall equipment costs.

#### 3.2.4. Microwave-Assisted Extraction

**Figure 5:**
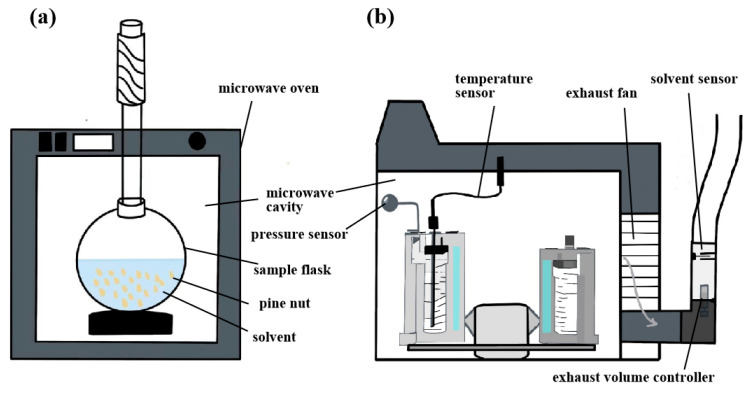
Open microwave-assisted extraction device (**a**) and closed microwave-assisted extraction device (**b**). (The effect of MAE is significantly affected by microwave power and heating time).

Over the past two decades, microwave-assisted extraction (MAE) has gained prominence in the extraction of plant-derived compounds [56]. MAE combines microwave irradiation with solvent extraction [53], significantly reducing solvent consumption and improving extraction efficiency. The technique offers several advantages, including simple equipment, low operational cost, high efficiency, and environmental sustainability [53,58]. A schematic of the microwave-assisted extraction device is presented in Figure 5. MAE uses an electromagnetic field to accelerate energy transfer, resulting in rapid and uniform heating of both the solvent and the matrix [58,59]. The combined effect of microwave heating and the disruption of cellular structures significantly enhances extraction efficiency [53,55]. In a typical procedure, tiger nut powder and a mixture of petroleum ether and acetone (2:1, *V*/*V*) were added to a flask. The flask was placed in a microwave oven cavity and connected to a cooling system. After extraction, the solvent was removed using a rotary evaporator at 50 °C under reduced pressure. An oil yield of 24.12% was obtained under the following conditions: microwave power of 420 W, temperature of 75 °C, a liquid-to-solid ratio 7.0 mL/g, and extraction time of 55 min. Although MAE resulted in a lower oil yield compared to SE, the contents of total phenols (92.53 mg GAE/kg), α-tocopherol (315.27 mg/kg), β-carotene (25.43 mg/kg), and phytosterols (685.68 mg/kg) in MAE-extracted oil were higher than those in SE oil [53]. Additionally, the FA composition was similar to that of SE oil [69]. MAE promotes the release of bioactive compounds such as tocopherols by disrupting lipoprotein membranes and breaking their associations with other components. Compared with conventional solvent extraction, MAE may also induce the Maillard reaction [69], which further enhances the antioxidant capacity and stability of the oil [53,55]. However, MAE is not suitable for thermally sensitive components, especially under prolonged exposure [55]. Lipid oxidation may occur due to the formation of both primary and secondary oxidation products [31,58,70]. In addition, the efficiency of MAE is significantly affected by microwave power and heating duration. The thermal effect generated by MAE accelerates the evaporation of water, which increases intracellular permeability. However, if the treatment time exceeds a certain threshold, the cell structure of oilseeds loses its plasticity and elasticity. This results in excessive fragmentation and the blockage of oil channels by fine particles, ultimately hindering the extraction of PNO [69]. The quality of oils is strongly influenced by microwave parameters. Excessive power or prolonged treatment can degrade bioactive compounds, including chlorophyll, carotenoids, phenols, and tocopherols [70]. It may also disrupt the double-bond structure of tocopherols. Moreover, uneven heating and over-processing of oilseeds may result in degradation, thereby reducing oil quality and yield [69].

In industrial applications, existing equipment often fails to meet the production requirements of nutritional health products and pharmaceuticals [71]. To address the issue, low-temperature microwave extraction has been proposed as a strategy to balance extraction efficiency and oil quality, with the goal of minimizing lipid peroxidation [44]. However, no studies have yet reported the extraction of PNO using microwave techniques. Future research should focus on optimizing MAE parameters, exploring its potential for PNO production, and comprehensively evaluating its economic feasibility and operational practicality.

#### 3.2.5. Supercritical Fluid Extraction

**Figure 6:**
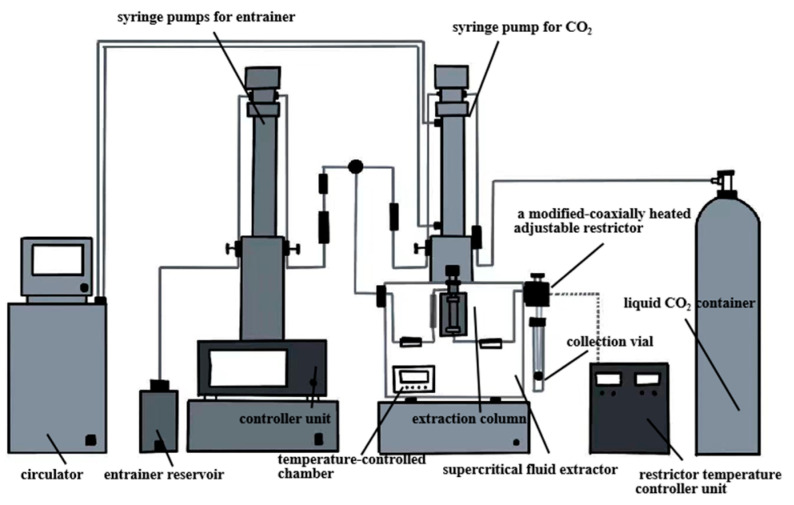
Supercritical fluid extraction device. (Extraction pressure and extraction time are the main factors affecting the extraction rate of PNO).

Supercritical fluid extraction (SFE) has garnered significant attention in the food and nutritional health product industries due to its high efficiency and environmentally friendly characteristics [54,55,59]. A supercritical fluid (SCF) is a unique phase of matter that exists above its critical temperature and pressure, where liquid and gas phases are indistinguishable. This state is particularly suitable for extraction processes [55]. Carbon dioxide is widely used as an SCF due to its inert nature, non-toxicity, recyclability, and low cost [57,59]. The apparatus used for SFE is shown in Figure 6. The high selectivity of the SCF is attributed to their tunable density [56]. The addition of a co-solvent further improves the precise extraction of target compounds [54]. Its high diffusion coefficient, low viscosity, and low surface tension enhance the penetration of solid matrices and increase extraction efficiency [59]. SFE is carried out at low temperature and in the absence of oxygen and light. These conditions effectively protect compounds that are heat-sensitive, have high boiling points, or are prone to oxidation [54]. Moreover, minimal solvent residue makes SFE an environmentally friendly extraction method [55].

In the supercritical extraction of PNO, extraction pressure and extraction time are the primary factors influencing yield [31]. As pressure increases, the density of supercritical CO_2_ also increases, enhancing oil solubility and thereby improving the recovery rate. However, extraction time must be optimized to balance efficiency and economic feasibility. While longer extraction times can enhance solute recovery, excessive durations may lead to higher labor costs, reduced equipment efficiency, and the co-extraction of undesirable impurities [57]. Optimization experiments indicated that a PNO yield of 45.85% was achieved under conditions of 5760.83 PSI, 50 °C, and 3.0 h [31]. Additionally, the oil obtained by this method was rich in α-linolenic acid, total phenols, and flavonoids. These compounds showed greater biological activity compared to oils extracted using conventional solvents [54]. Notably, the unsaturated fatty acid content of PNO obtained through supercritical extraction (89.20%) was significantly higher than that extracted using n-hexane via SE (87.17%) [72]. However, the high content of UFA poses challenges to oxidative stability. Inadequate deoxygenation, elevated temperatures, or high pressures can accelerate oxidative reactions and increase the risk of degradation.

Despite the clear technological advantages of SFE, its commercial application is limited by high operational costs, elevated working pressures, the absence of pilot-scale facilities, and complexity of modeling and scaling-up processes [54,56,57]. To meet industrial-scale production requirements, detailed studies on scale-up procedures are needed [55]. However, the development of SFE remains constrained by the lack of pilot-scale equipment, reliable modeling and scaling standards, and sufficient financial support [59].

### 3.3. Storage Conditions for PNO

Proper storage is essential for processing companies, particularly for high-quality products such as PNO. High-quality oils must be stored under strictly controlled conditions to prevent oxidation and rancidity, the primary causes of quality deterioration [12,73]. Therefore, it is necessary to optimize storage conditions to maintain the quality of PNO during long-term storage.

Oil stability is affected by multiple factors, including FA composition, phytonutrients, processing quality, temperature, light exposure, packaging materials, and oxygen concentration [38,51,74]. Storage conditions are particularly critical in hot climate [73]. Table 3 summarizes the key factors affecting PNO storage and presents corresponding countermeasures aimed at maintaining long-term quality and safety, preventing fungal and toxin contamination, and enhancing product competitiveness.

Lipid oxidation is a widespread chemical process that accelerates significantly with increasing temperature [75]. It follows a classical autooxidation mechanism, primarily initiated by free radicals generated from UFA [73]. In the case of PNO, exposure to high temperatures (>35 °C) markedly accelerates lipid oxidation. It increases PV, promotes browning, and doubles the oxidation rate for every 10 °C rise [33,38]. It also influences the formation of secondary oxidation products [73]. Phenolic compounds and flavonoids are also susceptible to degradation at elevated temperatures [38,39]. The PUFA content in soybean oil was 77.26% at 100 °C but decreased to 72.72% at 200 °C. Meanwhile, α-dicarbonyl compounds increased by approximately 15-fold [76], posing a potential risk to human health.

Low-temperature conditions slow the chemical kinetics of oil degradation [77]. Therefore, storing oils at 4 °C has proven effective in inhibiting these reactions [78]. However, storage methods such as refrigeration, freezing, and argon treatment do not significantly alter the overall FA profiles. However, they do affect the levels of PUFAs and saturated fatty acids (SFAs), possibly due to selective double bond cleavage. This provides further evidence of temperature’s influence on oil degradation [78]. Under cold conditions, oxygen solubility may increase. PUFAs are more mobile than SFAs, and higher unsaturation typically results in lower oxidative stability [79]. When temperature fluctuates, the energy required for oxidation becomes more available, increasing the risk of lipid oxidation. Therefore, storage temperature should remain as stable as possible. Maintaining this stability requires a balance between cold chain costs and the risks associated with temperature fluctuations [15,37,77].

The impact of light on the quality and stability of PNO is significant and should not be overlooked. Light accelerates peroxide formation in cooking oils through photooxidation processes [79,80]. This effect is further enhanced at elevated temperatures due to a reduction in activation energy [79]. The PV of olive oil stored at room temperature under light reached 8.2 meq O_2_/kg. This value was higher than those observed under dark conditions at 23 °C, 30 °C, and 40 °C, which were 5.9, 5.3, and 4.4 meq O_2_/kg. These findings suggest that photooxidation is more effective than autooxidation in generating hydrogen peroxide [73,79]. Under light exposure at room temperature, photoactivated chlorophyll reacts with UFA to form hydroperoxides. In addition, light promotes the decomposition of hydrogen peroxide, leading to the formation of secondary oxidation products such as aldehydes and ketones. These compounds further compromise the quality of cooking oils [73].

Comprehensive photoprotection strategies are essential to prevent photooxidative degradation. First, amber glass bottles with light-blocking properties are recommended, as they effectively prevent light-induced photochemical reactions [43]. Second, exposure time should be strictly controlled to minimize prolonged light contact [80]. Chemically, antioxidants may be added to inhibit photooxidative degradation. For example, 0.45 mg of flavonoids can reduce singlet oxygen-mediated oleic acid oxidation by 11.6% [81]. α-Tocopherol and carotenoids have also been shown to effectively protect vegetable oils [43]. However, the application of this strategy in the food industry requires caution. Flavonoids may introduce bitterness and astringency, alter sensory properties, and disrupt colloidal stability. Additionally, the extraction and purification processes are complex, which may limit their economic feasibility for large-scale use [82].

**Table 3:** PNO storage stability challenge: study on environmental factors and protection measures.

	Mechanism of Damage to PNO	The Solution	References
Light	Photo-induced oxidation accelerates the formation of pungent odor in PNO.	Darkroom storage, light-proof packaging, and the use of materials that block specific wavelengths of light (UV barrier film).	[43,79,80]
Oxygen	A free radical chain reaction is initiated, accelerating PNO oxidation and leading to the formation of unpleasant odors.	High-barrier packaging materials, nitrogen replacement, and vacuum sealing.	[15,83,84]
Temperature	When the temperature exceeded 35 °C, the stability of FA in PNO declined significantly, and the oxidation rate nearly doubled with every 10 °C increase.	Precise temperature control equipment to reduce temperature fluctuations.	[33,77,78]

### 3.4. Packaging Technology for PNO

The storage environment, such as low temperature and darkness, provides the foundation for oil protection. Packaging design further enhances the physical effectiveness of these storage conditions [15,83]. Together, they form an integrated system for maintaining PNO quality.

Oxygen concentration directly influences the risk of lipid oxidation and fungal contamination. High oxygen levels promote both processes. Therefore, establishing a stable anaerobic environment is essential for effective storage [84]. To achieve this, oxygen infiltration must be minimized through the use of airtight containers and the maintenance of stable humidity. Controlling humidity fluctuations is also critical to prevent hydrolysis and acidification caused by excess moisture [75,85]. However, ensuring complete removal of internal oxygen and preventing external oxygen ingress during packaging remains a technical challenge. As many food spoilage processes rely on oxygen and redox potential, techniques such as reducing headspace, using oxygen barrier materials, nitrogen flushing, or vacuum packaging are effective in minimizing oxidation risk [33,75,77]. Reduced brightness in oil samples has been associated with the presence of oxygen inside the packaging. Oils stored in a nitrogen atmosphere exhibit less reduction in L* (brightness). However, increases in a* (redness) and b* (yellowness) suggest degradation of natural pigments [86].

Although these techniques effectively limit oxygen exposure, concerns persist regarding residual oxygen during packaging and its possible ingress from external sources [14]. Appropriate packaging materials play a crucial role in maintaining food quality and extending shelf life [37,42]. Materials such as colored glass, plastics, tinplate, and UV-blocking PET provide inert barrier functions that significantly extend the shelf life of PNO [86]. However, materials prone to oxidative degradation, such as certain metal containers, should be avoided [87,88]. The bitterness intensity of olive oil stored in tin cans at 26 °C declined significantly, reaching 4.24 ± 0.13 after 125 days. A noticeable odor was also detected, indicating rapid quality deterioration. In addition, the content of volatile compounds such as (E)-2-hexenal also declined, adversely affecting the flavor profile of olive oil [87]. Although ceramic and glass packaging can slow oxidation by adsorbing hydroperoxides and polar compounds on hydrophilic surfaces, they may also accelerate hydroperoxide decomposition and increase volatile release. Therefore, such materials may be unsuitable for storing oils with important flavor attributes [88]. Nevertheless, even with high barrier performance, oil oxidation remains inevitable and can result in the formation of harmful oxidation products.

Currently, research on PNO remains limited. Future studies should focus on its fundamental properties. In addition, attention should be given to the direct influence of packaging technologies and storage conditions on the oxidation rate of PNO. A deeper understanding of these effects will help clarify the oxidation mechanisms of PNO. Moreover, the feasibility and limitations of these technologies under real-world application scenarios must be carefully evaluated. Additionally, it is essential to consider cost-effectiveness, environmental impact, and consumer preferences when designing optimized packaging and storage strategies.

## 4. Technological Approaches to Improve PNO Stability

Certain emerging technologies provide novel approaches for stabilizing PNO (Table 4). These techniques not only enhance its antioxidant properties but also expand its potential applications in the food industry.

### 4.1. Antioxidant

Antioxidants are compounds that prevent or slow lipid oxidation. They are essential for maintaining the freshness and quality of foods by inhibiting oxidative deterioration [95]. Growing health awareness has raised concerns about synthetic antioxidants such as butylated hydroxyanisole (BHA) and butylated hydroxytoluene (BHT). Despite their low cost and wide availability [18], these compounds are questioned due to potential toxicity and carcinogenic risks. Public health authorities and consumers increasingly prefer processed foods with more PUFAs and fewer additives [96]. As a result, plant extracts have attracted significant attention as sources of natural antioxidants. These extracts are obtained from natural sources such as fruits and vegetables [97]. They are rich in bioactive compounds including polyphenols, carotenoids, tannins, lignans, tocopherols, phenolic acids, and vitamins [14,17]. These natural antioxidants not only exhibit antioxidant activity comparable to synthetic compounds but also offer additional health benefits [18,51]. Natural antioxidants protect oils by autooxidation. They are key factors influencing the oxidative stability, isomerization rate, and free radical scavenging activity of PNO [90]. Among them, phenolic compounds are particularly effective in preventing lipid oxidation [77]. These compounds enhance the oxidative and thermal stability of vegetable oils by scavenging of free radicals and inhibiting oxidation reactions [18,52,98]. This aligns with modern consumer preferences for health, safety, and environmental sustainability. A comparison of the advantages and disadvantages of various antioxidants is presented in Table 5.

The distribution of antioxidants within colloidal systems significantly influences their efficacy. Polar antioxidants scavenge free radicals in the aqueous phase and inhibit the initiation of oxidation chain reactions. They also form a protective layer at the oil-water interface, thereby reducing the migration of oxidized substances from the aqueous phase to the oil phase. This polarity-dependent distribution determines the site of action and effectiveness of antioxidants in colloidal systems. Therefore, the selection of antioxidants for colloidal systems should consider the compatibility between their polarity and the system’s phase characteristics [99].

Wastes from the global food industry and by-products of fruit and vegetable processing serve as valuable sources for natural antioxidant extraction. These low-cost and sustainable antioxidants show great potential for applications in the food industry [17,98,100]. Studies have demonstrated that carnosic acid (CA) is more effective in inhibiting PNO oxidation compared to other natural and synthetic antioxidants, including vitamin E, BHA, and BHT. After 15 days of storage at 60 °C, the PV of 0.05 mg/g CA was similar to that of 0.2 mg/g BHT. The lowest PV was observed in the 0.2 mg/g CA group (91.12 ± 4.10 meq/kg), significantly lower than in samples containing BHT or VE [89]. Plant-based feed additives serve as natural alternatives in poultry nutrition. They not only improve feed quality but also meet consumer demand for natural ingredients [101].

However, several studies have reported limitations in the use of antioxidants. The addition of the mixed antioxidants (β-carotene-VE and astaxanthin-VE) to chia seed oil may reduce its oxidative stability. After 8 weeks of storage at 4 °C, the PV exceeded 150 meq/kg for the β-carotene component, while it remained below 10 meq/kg for the other components [102]. This effect may result from naturally occurring carotenoid promoting oxidation when combined with tocopherols, which are naturally present in chia seed oil [103]. Furthermore, combinations with more hydrophilic compounds (p-coumaric acid/β-carotene = 9:1) exhibited antioxidant synergy at 3.0 μm (SR = 22.33). In contrast, combinations with more lipophilic compounds (p-coumaric acid/β-carotene = 1:9) resulted in antioxidant antagonism (SR = 11.68). Therefore, the combination of antioxidants should be approached with caution. Careful selection and optimization are essential to maximize efficacy and maintain the nutritional value and quality of the food products [20].

**Table 5:** Types of antioxidants and comparison of their advantages and disadvantages.

Types		Advantages	Disadvantages	References
Synthetic antioxidants		Low cost, high stability, high antioxidant efficiency.	Potential toxicity, cancer risk controversy, low consumer acceptance.	[18]
Natural antioxidants	Polyphenols	High safety, antioxidant capacity, and provide additional health benefits.	The extraction process is costly, the concentration requires further optimization, and it may have antagonistic effect with other components.	[77]
	Carotenoid	Natural source, with pigment function.	High photosensitivity may promote oxidation of tocopherols.	[20,103]
	Tocopherol	Fat-soluble antioxidant to protect the oil phase from oxidation with high safety.	Single applications have limited effect and may fail at high concentrations.	[99,103]

### 4.2. Pickering Emulsions

Pickering emulsions form a mechanical barrier at the oil–water interface via solid particles. This barrier inhibits oil droplet coalescence and delays lipid oxidation. Unlike conventional surfactants, the stability of Pickering emulsions relies on the physical isolation provided by particles. This includes reduced interfacial tension and increased steric hindrance [104,105]. Whey protein–polyphenol composite particles increased interfacial protein content by 80.33% through crosslinking. They formed a thicker interfacial film and enhanced steric hindrance. After 14 days, the PV of emulsions containing tannic acid was 7.22 meq/kg, significantly lower than the 39.10 meq/kg in emulsions without tannic acid. The physicochemical stability of pine nut oil emulsions was significantly improved. Zein/apple pectin (5:3) composite particles can efficiently adsorb at the oil–water interface to form a dense and uniform interfacial layer. This structure inhibits oil droplet aggregation and improves interfacial properties. In addition, incorporating myricetin, a potent antioxidant, into the coating emulsion can significantly delay lipid oxidation. After 8 h of UV radiation, emulsions containing myricetin showed significantly lower malondialdehyde levels compared to those without myricetin [95]. Luteolin micro/nanoparticles also demonstrated potential in inhibiting lipid peroxidation, effectively preventing PNO oxidation [21]. However, the antioxidant efficacy of Pickering emulsions remains controversial. Although the interfacial barrier can delay oxidation, the size and distribution of Pickering particles limit their uniformity at the nanoscale. This limitation affects their ability to form a consistent protective barrier. As a result, small prooxidants, such as metal ions or free radicals, may still penetrate the particle layer and reach the lipid interface. After 72 h, the concentration of conjugated diene hydroperoxides in the Pickering emulsions reached 225 mmol/kg oil, which was not significantly different from that in conventional emulsions (125 mmol/kg oil) (*p* < 0.05) [106].

In addition, food-grade biopolymers, such as proteins and polysaccharides, are gradually replacing inorganic particles like SiO_2_ and TiO_2_ to meet safety standards, in response to growing health awareness [105,107]. Despite the emphasis on green and safe materials, the application of Pickering particles in the food industry remains limited, as not all particle types are approved for food use. Therefore, recent research has focused on identifying suitable existing particles that can function as Pickering stabilizers [95]. However, the potential allergenicity of some biopolymer-based particles, such as casein and gliadin, may restrict their practical application [107].

### 4.3. Microencapsulation

Microencapsulation is a technique in which the core material is enclosed within one or more materials, such as a shell, polymer matrix, or wall material, to form a protective barrier. This barrier shields the core from biotic and abiotic stressors and helps preserve its biological, functional, and physicochemical properties [74,108]. Encapsulation technology is widely applied in the food, agriculture, and pharmaceutical industries, especially for protecting oils from degradation [13,97]. This technology enhances the oxidative and thermal stability, as well as the shelf life of oils, by stabilizing emulsions and encapsulated structures [12,74]. These structures create a protective layer that enables the effective storage of bioactive compounds [109]. Microencapsulation is valued for its unique advantages. It can convert liquid oils into solid forms [100], enriching the diversity and nutritional value of food products. Additionally, it reduces the unpleasant sensory properties and volatility of oils [13,110], making them more suitable for use in various food products, such as in bread, yogurt, milk, and infant formulas [111].

Maillard reaction product was prepared using gelatin, gum arabic, and maltodextrin (2:2:1, W:W:W) as well materials. Microcapsules were formed through complex coacervation and freeze-drying. The results showed that microencapsulated PNO exhibited greater oxidative stability compared to unencapsulated PNO [91]. PNO microencapsulated powder has been successfully developed using microencapsulation technology by stabilizing the PNO emulsion and selecting appropriate wall materials [12]. The choice of wall material is critical to the oxidative stability of encapsulated oils [100]. Higher encapsulation efficiency contributes to improved oxidative resistance. However, during large-scale emulsion preparation, phase separation and high oil loading can increase surface oil content, thereby elevating the risk of oxidation [12,20]. Therefore, selecting wall materials with suitable molecular weights and optimized combinations is essential to maintaining the stability and antioxidant performance of microcapsules [36]. Maillard reaction products, when used as antioxidant wall materials, can further improve the antioxidant properties of microcapsules under specific conditions [91]. With the advancement of microencapsulation technology, novel green materials—such as fibers and proteins derived from food waste and by-products—have emerged. These materials not only provide sustainable encapsulating agents but also offer additional health benefits [100]. Although microencapsulation technology holds great promise in the food industry, concerns regarding the safety, regulatory approval, and bioactivity of encapsulated compounds require further investigation. Microencapsulation plays a vital role in the development of functional foods. However, comprehensive studies on the toxicity and bioactivity of micro- and nanoparticles are essential to ensure the safe, effective, and functional delivery of bioactive compounds [36,110]. Processing conditions, such as high temperatures during spray drying and oxygen exposure, significantly affect the oxidative stability of oils [100]. Optimization of wall materials and processing parameters allows for microencapsulation technology to deliver safe, high-quality oils and functional foods to the industry.

### 4.4. Oleogel

**Figure 7:**
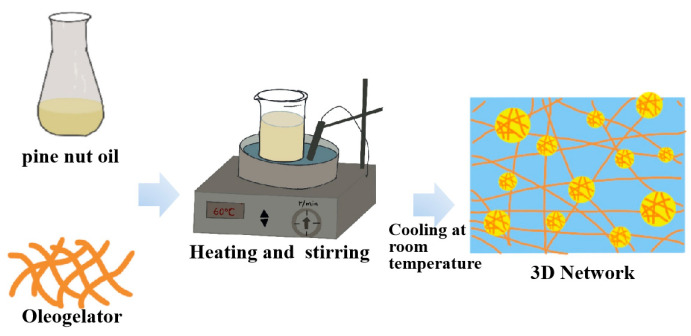
Preparation of PNO oleogel.

Oil gelation technology is an emerging approach in both research and industry [112]. It aims to transform liquid oils into low-saturated, trans-fat-free solid fat substitutes, thereby improving the functional properties and nutritional value of food products [94]. Vegetable oils such as PNO, which are rich in UFA and bioactive compounds [4,92], can be converted into solid gels using oleogelators (Figure 7) [93]. These oleogels retain the nutritional properties of the oils and significantly enhance the texture, thermal stability, and antioxidant capacity of the final product [92]. Oleogels form a three-dimensional crystalline gel network that increases the oil viscosity and restricts the mobility of oil molecules. This structure reduces direct contact between oxygen and oil, thereby delaying oxidative reactions. Some oleogels also possess intrinsic antioxidant radicals [113,114]. This technology provides new opportunities for the development of healthier food products, such as low-fat mayonnaise, plant-based meat alternatives, and ice cream. It also offers a sustainable solution to the cocoa butter shortage in the chocolate industry [115]. All oleogel samples exhibited higher PV than fresh oil prior to storage. This may be due to the exposure of oils to high temperatures during the oleogelation process. However, after storage, their PVs were lower than those of pure oil. These findings highlight the critical role of the gel network in inhibiting oil oxidation. The dense structure of oleogels significantly limits oxygen diffusion and penetration into the matrix [113]. It has also been demonstrated that the peroxide value of all oleogel samples are lower than those of pure macadamia oil [114].

Due to their high customizability, oleogel can be formulated and processed on demand to optimize properties such as rheology, oil retention, and stability. This enables improved shelf life and overall product stability. Additionally, oleogel can serve as carriers for bioactive compounds [93,116], thereby enhancing the health value of food products [94]. However, further toxicological studies are needed to evaluate the digestive behavior and safety of oleogel, which essential for improving consumer acceptance and regulatory approval.

Although oleogel technology holds significant potential [116], its commercialization still faces several challenges. These include the limited availability of food-grade and cost-effective gelling agents, compatibility with food systems, and processing stability [94]. In particular, lipid oxidation remains a critical issue that must be addressed by optimizing parameters such as heat treatment [92,93]. Current research aims to enhance both the sensory attributes and shelf life of oleogels. Efforts are being made to improve formulations and processing conditions to better mimic the taste and texture of traditional fats while maintaining the associated health benefits. The sensory characteristics of oleogels, including taste and texture, differ from those of traditional fat products. These differences may influence consumer acceptance [117].

Olive oil-based oleogels have been shown to extend the shelf life of sponge cakes [118]. They also improve cake fluffiness by promoting the formation of a dense gluten network and reducing water loss [119]. Most existing studies on oleogels in bakery products focus on end-product characterization. However, there is a lack of comprehensive comparisons between different types of oleogels and traditional solid fats, particularly in terms of functional properties and application scenarios [120]. One major concern is the significantly higher production cost of oleogels compared to traditional oils, which places the final products at a disadvantage in price competitiveness. This limits their widespread application in the food industry and reduces consumer acceptance.

## 5. Conclusions and Future Perspectives

PNO contains high concentrations of distinctive UFA, conferring significant nutritional and health benefits. However, lipid oxidation during extraction and storage remains a major challenge. Oxidation not only compromises product quality and safety but also reduces its nutritional value. Therefore, identifying and summarizing the causes of PNO oxidation throughout processing and storage is essential. The food industry is actively optimizing pretreatment processes to enhance the flavor and storage stability of PNO. Green technologies such as microwave- and ultrasound-assisted extraction are being explored to improve efficiency and reduce environmental impact. However, these techniques still expose PNO to a risk of oxidation. To address storage-related oxidation, strategies such as light-proof packaging, oxygen reduction technology, and temperature control have been applied to extend shelf life and preserve the nutritional quality of PNO. Nevertheless, the high oxidative susceptibility of UFA remains a significant challenge.

A range of new technologies is currently being developed to reduce the oxidation of PNO. Researchers are actively developing natural antioxidants, particularly those derived from agricultural wastes and other potential resources. In parallel, advanced technologies such as Pickering emulsions, microencapsulation, and oleogel systems are making continuous progress, offering novel strategies to protect oxidation-sensitive components in PNO. Although these technologies exhibit significant application potential, their practical implementation still encounters several challenges. Key barriers to the large-scale adoption of these technologies include the potential allergenicity of some novel carrier materials, the absence of a comprehensive long-term safety evaluation system for bioactive compounds, and the increased costs arising from complex preparation processes. Future research should focus on breakthroughs in the three key areas, efficacy verification, safety assessment, and cost control, to advance these technologies towards large-scale application.

## Figures and Tables

**Table 4:** Methods for improving PNO oxidation stability: mechanisms, advantages, disadvantages, and applications.

Methodologies	Mechanism of Action	Advantages	Disadvantages	Application in PNO	References
Antioxidant	Scavenging free radical to inhibit oxidation reaction	Reduce the rate of oxidation and provide health benefits.	The use of antioxidants in combination with PNO may pose a risk of reduced oxidative stability.	PNO supplemented with 0.2 mg/g carnosic acid showed a favorable oxidative effect.	[89,90]
Pickering emulsions	Solid particles form a mechanical barrier at the oil–water interface, which physically isolates the oil and thereby slows its oxidation	Increased interfacial protein content, enhanced rheological properties, and improved protection during digestion.	With potential toxicity and allergy, small-molecule prooxidants can still penetrate the granular layer.	Luteolin micro/nanoparticles can serve as stabilizers. They not only maintain the structural integrity of emulsion droplets but also enhance the oxidative stability of PNO emulsions.	[21]
Microencapsulation	Forms a protective layer to store biologically active substances	Converts liquids to solids, enriches food range improves oxidative stability and shelf life.	Toxicity and biological activity of micro- and nanoparticles.	PNO microcapsules were prepared using gelatin– gum arabic–maltodextrin (2:2:1, *w*/*w*) as the Maillard reaction-based wall material.	[74,91]
Oleogel	Physical isolation reduces the amount of oil molecules in the environment	Low-saturated, trans-fat-free health product, efficient carrier of bioactives.	Processing technology requirements are high; there are food compatibility problems.	\	[92,93,94]

## Data Availability

All data supporting the findings of this study are available within the manuscript.

## Abbreviations

The following abbreviations are used in this manuscript:

PNO	Pine nut oil
MAE	Microwave-assisted extraction
UAE	Ultrasonic-assisted extraction
FA	Fatty acid
MUFA	Monounsaturated fatty acid
PUFA	Polyunsaturated fatty acid
UFA	Unsaturated fatty acid
TV	Totox value
AV	Acid value
PV	Peroxide value
SE	Soxhlet extraction
BHT	Butylated hydroxytoluene
BHA	Butylated hydroxyanisole
SCF	Supercritical fluid
SFE	Supercritical fluid extraction
CA	Carnosic acid
DPPH	1,1-Diphenyl-2-picrylhydrazyl
GAE	Gallic acid equivalent
